# Supplementary material for: The impact of role overload on healthcare workers’ psychological distress and well-being: The mediating role of task distraction and moderating roles of resilience and emotional intelligence
Source: PLOS Ment Health. 2025 Dec 10;2(12):e0000461. doi: 10.1371/journal.pmen.0000461 (PMC12798228; doi:10.1371/journal.pmen.0000461)
Supplement: S1 Questionnaire — (DOCX) [file pmen.0000461.s002.docx]

**Research Questionnaire**

Dear Respondent!

I am a research scholar. I am working on a research article entitled **“Impact of Role Overload on Psychological Distress and Well-Being: A Mediation and Moderation Analysis”** This study aims to explore the impact of role overload (RO) on psychological distress and well-being among healthcare professionals, examining the mediating role of task distraction (TD) and the moderating roles of employee resilience (ER) and emotional intelligence (EI). Your answers will be kept strictly confidential and will be used only for research purpose only. Your identity will be not disclosed on this document so kindly give an honest opinion to make this research unbiased.

You are requested to take 5 minutes out of your busy schedule to fill this questionnaire. Although you are not bound to answer these questions and at any point in time, you can quit answering but still I will be privileged by your opinion in this research work. If you need findings of this research, please order a copy at [fatimanoreen1122@gmail.com](mailto:fatimanoreen1122@gmail.com)

Once again thanks for your precious time and cooperation

Regards,

Fatima Noreen

Research Scholar

This study aims to explore the impact of role overload (RO) on psychological distress and well-being among healthcare professionals, examining the mediating role of task distraction (TD) and the moderating roles of employee resilience (ER) and emotional intelligence (EI).

| **SECTION A: PERSONAL INFORMATION** |
| --- |
| Your Name: (Optional) |
| Your gender: 1. Male 2. Female |
| Your Education: 1. Matric 2. Intermediate 3. Graduation 4. Masters 5. MS/Phil |
| Working experience (in years): |
| Your age (in years, like 40 years) |

Please tick what is most appropriate to you

| **Strongly Disagree** | **Disagree** | **Not Sure** | **Agree** | **Strongly Agree** |
| --- | --- | --- | --- | --- |
| 1 | 2 | 3 | 4 | 5 |

| **SECTION B:ROLE OVERLOAD** | | | | | | |
| --- | --- | --- | --- | --- | --- | --- |
| 1 | There is a need to reduce some parts of my role | 1 | 2 | 3 | 4 | 5 |
| 2 | I feel overburdened in my role | 1 | 2 | 3 | 4 | 5 |
| 3 | I have been given too much responsibility | 1 | 2 | 3 | 4 | 5 |
| 4 | I amount of work I have to do interferes with the quality I want to maintain | 1 | 2 | 3 | 4 | 5 |
| 5 | My workload is too heavy | 1 | 2 | 3 | 4 | 5 |
| *Peterson, M. F. , Smith, P. B. , Akande, A. , Ayestaran, S. , Bochner, S. , Callan, V. , Cho, N. G. , Jesuino, J. C. , D'Amorim, M. , & Francois, P. H. (1995). Role conflict, ambiguity, and overload: A 21‐nation study. Academy of Management Journal, 38(2), 429–452* | | | | | | |

| **SECTION C:EMPLOYEE RESILIENCE** | | | | | | |
| --- | --- | --- | --- | --- | --- | --- |
| 1 | I tend to bounce back quickly after hard times | 1 | 2 | 3 | 4 | 5 |
| 2 | I have a hard time making it through stressful events | 1 | 2 | 3 | 4 | 5 |
| 3 | It does not take me long to recover from a stressful event. | 1 | 2 | 3 | 4 | 5 |
| 4 | It is hard for me to snap back when something bad happens | 1 | 2 | 3 | 4 | 5 |
| 5 | I usually come through difficult times with little trouble | 1 | 2 | 3 | 4 | 5 |
| 6 | I tend to take a long time to get over set-backs in my life | 1 | 2 | 3 | 4 | 5 |
| *The Brief Resilience Scale Smith et al. (2008)* | | | | | | |

| **SECTION D:TASK DISTRACTION** | | | | | | |
| --- | --- | --- | --- | --- | --- | --- |
| 1 | “How often are you for some reason disturbed so that you do not get the opportunity to fully immerse yourself in the task you have in front of you? | 1 | 2 | 3 | 4 | 5 |
| 2 | “How often are you interrupted when you have little time to complete an important task?” | 1 | 2 | 3 | 4 | 5 |
| 3 | To what extent are you disturbed by colleagues’ conversations and phone calls? | 1 | 2 | 3 | 4 | 5 |
| 4 | “To what extent are you disturbed by the instantaneous sound from other colleagues’ ringtones, text message alerts, people walking, computers, etc | 1 | 2 | 3 | 4 | 5 |
| *A. Seddigh, E. Berntson, C. Bodin Danielsson, H. Westerlund (2014) Concentration requirements modify the effect of office type on indicators of health and performance*. *Journal of Environmental Psychology, 38 (2014), p. 167-174* | | | | | | |

| **SECTION E: PSYCHOLOGICAL DISTRESS** | | | | | | |
| --- | --- | --- | --- | --- | --- | --- |
| 1 | About how often did you feel nervous | 1 | 2 | 3 | 4 | 5 |
| 2 | About how often did you feel hopeless | 1 | 2 | 3 | 4 | 5 |
| 3 | About how often did you feel restless or fidgety? | 1 | 2 | 3 | 4 | 5 |
| 4 | How often did you feel so depressed that nothing could cheer you up? | 1 | 2 | 3 | 4 | 5 |
| 5 | About how often did you feel that everything was an effort? | 1 | 2 | 3 | 4 | 5 |
| 6 | About how often did you feel worthless? | 1 | 2 | 3 | 4 | 5 |
| Kessler et al. 2003 | | | | | | |

| **SECTION F:PSYCHOLOGICAL WELL BEING** | | | | | | |
| --- | --- | --- | --- | --- | --- | --- |
| 1 | No one really cares if I am dead or alive | 1 | 2 | 3 | 4 | 5 |
| 2 | I am often bored | 1 | 2 | 3 | 4 | 5 |
| 3 | It is exciting to be alive | 1 | 2 | 3 | 4 | 5 |
| 4 | Sometimes I wish that I never wake up | 1 | 2 | 3 | 4 | 5 |
| 5 | I feel that life is worth living | 1 | 2 | 3 | 4 | 5 |
| 6 | I don’t seem to care about what happens to me | 1 | 2 | 3 | 4 | 5 |
| Reker and Wong (1988) | | | | | | |

### Wong and Law Emotional Intelligence Scale (WLEIS)

**Instructions:**Please indicate the extent to which you agree or disagree with the following statements, using the scale below:

**1 = Strongly Disagree**
**2 = Disagree**
**3 = Slightly Disagree**
**4 = Neutral**
**5 = Slightly Agree**
**6 = Agree**
**7 = Strongly Agree**

**1. Self-Emotion Appraisal (SEA)**

1. I have a good understanding of my own emotions.
2. I really understand what I feel.
3. I always know whether or not I am happy.
4. I have a good sense of why I have certain feelings most of the time.

**2. Others’ Emotion Appraisal (OEA)**

1. I always know my friends’ emotions from their behavior.
2. I am a good observer of others’ emotions.
3. I am sensitive to the feelings and emotions of others.
4. I have good understanding of the emotions of people around me.

**3. Use of Emotion (UOE)**

1. I always set goals for myself and try my best to achieve them.
2. I always tell myself I am a competent person.
3. I am a self-motivating person.
4. I would always encourage myself to try my best.

**4. Regulation of Emotion (ROE)**

1. I am able to control my temper so that I can handle difficulties rationally.
2. I am quite capable of controlling my own emotions.
3. I can always calm down quickly when I am very angry.
4. I have good control of my own emotions.

Wong, C.-S., & Law, K. S. (2002). The effects of leader and follower emotional intelligence on performance and attitude: An exploratory study. *The Leadership Quarterly, 13*(3), 243–274. https://doi.org/10.1016/S1048-9843(02)00099-1
