# Supplementary material for: The impact of role overload on healthcare workers’ psychological distress and well-being: The mediating role of task distraction and moderating roles of resilience and emotional intelligence
Source: PLOS Ment Health. 2025 Dec 10;2(12):e0000461. doi: 10.1371/journal.pmen.0000461 (PMC12798228; doi:10.1371/journal.pmen.0000461)
Supplement: Attachment — Submitted filename: AUTHORS RESPONSE TO REVIEWERS PLOS MH.docx [file pmen.0000461.s004.docx]

**AUTHORS RESPONSE TO REVIEWERS**

Dear Editor,

First of all, we would really like to extend our sincere gratitude to you and the reviewers for acknowledging the efforts in terms of first revised version of our manuscript. The constructive feedback and comments by reviewers helped us a lot in improving the quality of the manuscript.

Secondly, we are thankful to the reviewers for evaluation and valuable comments on our manuscript titled “The Impact of Role Overload on Healthcare Workers’ Psychological Distress and Well-Being: The Mediating Role of Task Distraction and Moderating Roles of Resilience and Emotional Intelligence” submitted to *PLOS Mental Health*. The reviewer’s comments are repeated below in italics followed by our response and action. These responses/modification/additions are mentioned in red font in the manuscript. The complete revision in responding to the comments can be followed in the annotated version of the original manuscript.

We hope the revised version addresses a few shortcomings and strengthens the manuscript.

Thank you for your guidance and consideration.

Thank you,

The Authors

**Reviewer # 1: Dr. David Onchonga**

**Reviewers’ comment # 1: *Language and Grammar:***

*1.1 The manuscript contains frequent grammatical errors, awkward phrasing, and inconsistent sentence structure. For example:*

*“Data was collected from 600 healthcare workers, A cross-sectional design…” → should be split into two sentences.*

**Authors Reply**

We have thoroughly reviewed the manuscript for language clarity and corrected all grammatical errors, awkward phrasing, and sentence structure inconsistencies. Specifically, we made the following revision:

Data were collected from 600 healthcare workers (Physician, Nurses, Allied Health Professionals) working in both public and private healthcare sectors….. (Material and methods section, line111-113, page 6)

*1.2. “The scope of this study encapsulates quantitative in regard to a purposive sampling technique…” → unclear and needs rephrasing.*

**Authors Reply**

Noted and followed (Material and methods section, Line 115-117, page 6)

**Reviewers’ comment # 2 *Figures and Tables*** *:*

*Several figures are missing or improperly rendered. Ensure all figures are embedded correctly and labelled with captions.*

*Tables are informative but need consistent formatting and clearer titles. For example, Table 4 (Mediation Analysis) could benefit from clearer column headers and explanation of VAF.*

**Authors Reply**

We have re-embedded all figures and ensured proper formatting. For Table 4 (Mediation Regression Analysis), we revised the column headers for clarity and added an improved explanation of Variance Accounted For (VAF), which was previously unclear (Results section, line 231, page 14). Additionally, Table 6 has been revised to clarify the statistical reporting and language. Specifically, we changed wording such as “accepted/rejected” to “supported/not supported” to ensure accuracy in hypothesis testing (Results section, line 284, page 19-21).

**Reviewers’ comment # 3 *Data Availability:*** *The statement “data available within the manuscript” does not meet PLOS’s open data policy. Please upload the dataset to a public repository*

**Authors Reply**

We have updated the Data Availability Statement to comply with PLOS’s requirements. The “minimal data set” underlying the results is now available in the supplementary files submitted with this manuscript. Due to ethical restrictions, we have shared de-identified data set. Participants were given the assurance that their information would be kept private and would not be shared with anyone outside the research team during the consent procedure.

**Reviewers’ comment # 4 *Novelty and Contribution:***

*While the integration of emotional intelligence and resilience is valuable, the manuscript should better articulate its unique contribution compared to existing literature.*

*Consider emphasizing how your findings differ from or build upon prior studies, especially in the Pakistani healthcare context.*

**Authors Reply**

We have enhanced the discussion to highlight how integrating emotional intelligence (EI) and resilience as moderators in the relationship between role overload and psychological distress is a novel contribution. This study also fills a gap in the literature by focusing on healthcare workers in Pakistan, an underexplored context (Novelty and Contribution section, line 338-347, page 23).

**Reviewers’ comment # 5 *Discussion and Limitations*:**

*5.1 The discussion section reiterates results but lacks critical reflection. Please:* *Address limitations such as cross-sectional design, potential self-report bias, and cultural specificity. 358-368*

**Authors Reply**

As per instructions, we have expanded the Limitations section to address the cross-sectional design, self-report bias, and cultural specificity of the study (Discussion section, line 368-378, page 25)

*5.2 Discuss implications for policy and practice more deeply, especially regarding implementation of EI and resilience training.*

**Authors Reply**

Noted and followed (Discussion section, line 358-366, page 24).

**Reviewers’ comment # 6 *Formatting and Consistency:***

*Ensure consistent use of abbreviations (e.g., PD, PWB, RO) throughout the manuscript.*

*Reference formatting should follow PLOS guidelines. Some references are missing proper punctuation or DOI formatting.*

**Authors Reply**

We have standardized the use of abbreviations throughout the manuscript. The terms psychological distress (PD), psychological well-being (PWB), and role overload (RO) are now consistently used across the manuscript. Moreover, all references have been formatted correctly in Vancouver style and cross-checked with PLOS guidelines. Errors like capitalization and DOI formatting have been corrected.

**Reviewers’ comment # 7 *Ethical and Consent Documentation***

*While ethical approval is mentioned, please ensure that the supplementary documents (consent form and IRB approval) are properly referenced in the manuscript and uploaded as supporting information.*

**Authors Reply**

We have updated the manuscript to include references to the informed consent form and ethical approval documentation, which are now uploaded as supplementary materials. The manuscript includes the statement:

Ethical approval for this study was obtained from the Institutional Review Board (IRB) of the University of Lahore, Pakistan (Ref No: REC-UOL-/529/08/24). The informed consent form is available as supplementary material.

**Reviewer # 2: Anonymous**

*.*

***Reviewers’ comment # 1:***

*Need of the study should be revised.*

**Authors Reply**

We have revised the Introduction to better explain the need for this study, particularly in light of the increasing challenges faced by healthcare workers due to high workloads and stress (Introduction section, line 98-107, page 5)

***Reviewers’ comment # 2***

*Discussion section should be written in detail.*

**Authors Reply**

We have expanded the Discussion to provide more in-depth insights into the practical implications of our findings, how healthcare organizations can incorporate resilience-building programs and emotional intelligence (EI) training into their professional development initiatives to reduce stress and improve overall well-being among healthcare workers.

**Reviewers’ comment # 3**

*Rethink on limitations of the study and FUTURE RECOMMENDATIONS*

**Authors Reply**

We have revisited the Limitations section and provided more detailed insights into the study's limitations. Specifically, we discussed how the cross-sectional design limits the ability to establish causal relationships and highlight the need for longitudinal and experimental studies in the future.

**Editor’s Additional Comments**

**1***. At this time, please confirm that your submission contains your "minimal data set", which PLOS defines as consisting of the data set used to reach the conclusions drawn in the manuscript with related metadata and methods, and any additional data required to replicate the reported study findings in their entirety. This includes:

a) The values behind the means, standard deviations and other measures reported;

b) The values used to build graphs;

c) The points extracted from images for analysis*

We confirm that:
**(a)** The file *“Minimal Dataset HealthcareWorkers.xlsx”* contains all raw values underlying the reported means, standard deviations, and analyses, including item-level data and composite variables.
**(b)** The file includes all numerical values used to generate the manuscript figures (gender, profession, and experience distributions).

**(c)** No numeric data were extracted from images for analysis; all values derive from participant survey responses.

(The minimal dataset underlying all findings including raw item responses, computed composite scores, and the numerical values used to construct all figures and tables is provided in the Supporting Information file “Minimal Dataset HealthcareWorkers.xlsx.” The dataset contains de-identified participant-level data for Role Overload, Task Distraction, Employee Resilience, Psychological Distress, Psychological Well-Being, and Emotional Intelligence, together with demographic variables (gender, profession, experience, city, and hospital type). No numeric data were extracted from images for analysis. All data were collected under ethical approval from the Institutional Review Board of the University of Lahore, Pakistan (Ref No: REC-UOL-/529/08/24)

**2.** *Regression analyses: In tables, please report p-values as “<0.001” rather than “0.001.”*

**Authors Reply**

We have updated the p-value formatting across all tables, ensuring that values are presented as <0.001 as per the editor’s suggestion.

**3.** *Table 6: Hypotheses cannot be “accepted”; only the null hypothesis may be rejected. Please revise the language for statistical accuracy.*

**Authors Reply**

Noted and followed.

**4.** *Ensure in-text citations are accurate. In particular, when citing Role Conflict, Ambiguity, and Overload: A 21-Nation Study, please use the title exactly as published and do not alter it.*

**Authors Reply**

We have carefully reviewed the Methods section and ensured that all in-text citations are accurate, particularly the citation for Role Conflict, Ambiguity, and Overload: A 21-Nation Study, which is now referenced correctly.

**5**. *Causal vs. associational language: Please replace causal terms (e.g., affect, influence, increase) with associational terms (e.g., related to, covary with, associated with) to reflect the cross-sectional nature of your design.*

**Authors Reply**

We have revised the manuscript to replace causal terms with associational terms. For example:

•	This study affects…to….. This study is related to…

**6**. *Language and style: Please have a copy editor review the manuscript for English usage. For example, “this study utilizes” should begin a new sentence rather than follow a comma.*

**Authors Reply**

The manuscript has undergone a thorough copyediting process to improve readability, sentence structure, and overall flow. Changes include rephrasing awkward sentences and improving transitions between sections.

**Conclusion**

We believe the revisions made address all the points raised by the reviewers and the editor. The manuscript has been substantially improved and is now ready for further review. We are grateful for your time and consideration, and we look forward to your feedback.

**Sincerely,**
The Authors
